# Haemothorax and Empyema

**Published:** 1918-11

**Authors:** 


**Haemothorax and Empyema.** Lt. Col. Dobson, Brit. Med. Jour., Feb. 2, after performing pleurotomy at the site of election and evacuating the pleura, introduces a curved canula in the mammillary line in the 3d or 4th space, the canula ending blindly but having a dozen lateral openings. An antiseptic is introduced by a fountain or bulb syringe and drainage is effected through the drain behind, in the pleurotomy wound. J. Campbell, ibid. Jan. 26, fills the pleural cavity with Dakin's solution and withdraws it after 2 hours, repeating the process several times a day. Failure indicates a foreign body, a septic clot or the formation of an intrapulmonary abscess. He claims to have abridged the process of healing very considerably.

